# High-resolution whole-heart angiography with compressed sensing and 3D respiratory motion compensation in 5 minutes

**DOI:** 10.1186/1532-429X-17-S1-P36

**Published:** 2015-02-03

**Authors:** Mehdi Hedjazi Moghari, David Annese, Tal Geva, Andrew J Powell

**Affiliations:** 1 Cardiology, Boston Children's Hospital, Boston, MA, USA; 2 Pediatrics, Harvard Medical School, Boston, MA, USA

## Background

The electrocardiogram and respiratory-gated 3D steady-state free precession (3D-SSFP) sequence acquired during free-breathing generates a high-resolution anatomic datasets of the entire thorax, allowing for a comprehensive evaluation of intracardiac, coronary, and vascular abnormalities. An important limitation of 3D-SSFP, however, is its long imaging time during which the patient's heart rate, breathing pattern, and body position may change leading to reduced image quality or an incomplete scan. We, therefore, sought to reduce 3D-SSFP imaging time by combining 3D-LOC, a new respiratory motion compensation algorithm that improves scan efficiency [1], with compressed sensing (CS) reconstruction [2].

## Methods

**Figure 1:**
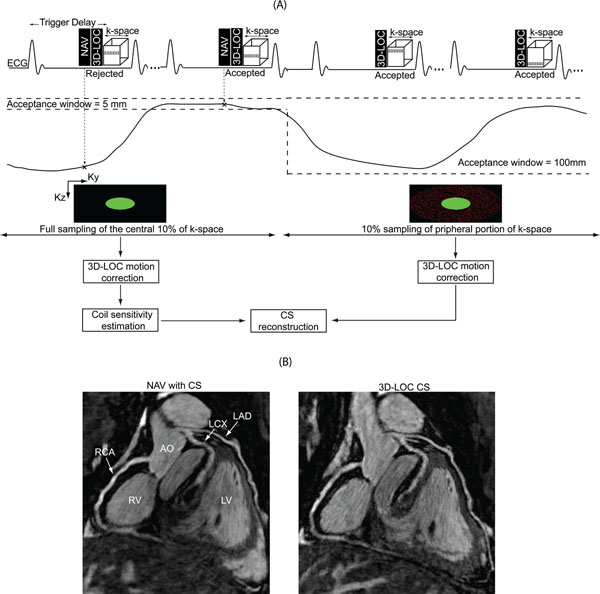
(A) Schematic diagram of the proposed 3D-LOC CS algorithm. (B) Coronal reformatted images from the same volunteer acquired with the NAV with CS and 3D-LOC CS techniques.

A schematic of the proposed 3D-LOC CS technique is shown in Figure 1A. The 3D-SSFP pulse sequence is immediately preceded by a conventional 1D navigator (NAV) and then the 3D-LOC which uses the startup pulses of the 3D-SSFP sequence to acquire a single-shot low-resolution 3D image. At the start of the scan, the central 10% of 3D-SSFP k-space is acquired using respiratory NAV gating. Then, the NAV window is widened resulting in 100% acceptance for the rest of the scan. During this time, 10% of the remaining peripheral 90% of 3D-SSFP k-space is randomly sampled to complete the scan. Offline, 3D-LOC data is used to correct the k-space data for the bulk respiratory motion of the heart in all 3 dimensions, and the CS-LOST reconstruction algorithm is used to estimate the unmeasured k-space data.

With IRB approval, 10 volunteers each underwent 2 3D-SSFP acquisitions: 1) conventional NAV with CS, and 2) the proposed 3D-LOC CS. For both, imaging parameters were FOV ~300×300×100 mm^3^, spatial resolution 1.0 mm^3^; α/TE/TR 70°/1.95/3.9 ms, bandwidth 0.64 kHz, k-space undersampling with a reduction factor of 5, and a 32-element receiver coil. Sharpness of the 3 coronary arteries was subjectively graded on a 4-point scale by 2 clinicians and objectively measured. Subjective and objective measures were compared using a signed-rank test and paired student t-test, respectively.

## Results

All acquisitions were successfully completed. Images from the same subject with the 2 3D-SSFP acquisitions are shown in Figure 1B. Results for all subjects are shown in Table 1. The scan time of the proposed 3D-LOC CS technique was significantly shorter than conventional NAV with CS (*p*<0.05). 3D-LOC CS had better objective vessel sharpness for all 3 coronary arteries (*p*<0.05), and there were no differences in subjective vessel sharpness for all 3 coronary arteries.

**Table 1:** Comparison of NAV with CS and 3D-LOC CS (n=10).

	NAV with CS	3D-LOC CS	p-value
Scan time (min)	6.3 ± 1.7	4.8 ± 1.1	<0.01

RCA subjective sharpness	3.35 ± 0.58	3.45 ± 0.60	1

RCA objective sharpness	0.52 ± 0.09	0.58 ± 0.09	0.01

LAD subjective sharpness	3.25 ± 0.72	3.35 ± 0.75	1

LAD objective sharpness	0.52 ± 0.12	0.56 ± 0.08	0.01

LCX subjective sharpness	3.05 ± 0.89	3.05 ± 0.89	1

LCX objective sharpness	0.49 ± 0.09	0.55 ± 0.09	0.01

Values are mean ± standard deviation. Subjective sharpness: 1-poor to 4-excellent. Objective sharpness: 0-blurred to 1-sharp. LAD = left anterior descending coronary artery; LCX = left circumflex coronary artery; RCA = right coronary artery.

## Conclusions

The improved respiratory gating efficiency of 3D-LOC combined with k-space undersampling and CS reconstruction achieved good quality 3D-SSFP images of the chest with 1 mm^3^ spatial resolution in a mean scan time of 5 minutes. Compared to conventional NAV, respiratory motion compensation with 3D-LOC yielded superior objective vessel sharpness in a shorter scan time.

## Funding

This work was supported by the Translational Research Program at Boston Children's Hospital, and by the Higgins Family Noninvasive Imaging Research Fund.
